# Restraining vocal fold vertical motion reduces source-filter interaction in a two-mass model

**DOI:** 10.1121/10.0025124

**Published:** 2024-03-01

**Authors:** Tsukasa Yoshinaga, Zhaoyan Zhang, Akiyoshi Iida

**Affiliations:** 1 Graduate School of Engineering Science, Osaka University, Toyonaka, Osaka 560-8531, Japan; 2 Department of Head and Neck Surgery, University of California, Los Angeles, Los Angeles, California 90095, USA; 3 Department of Mechanical Engineering, Toyohashi University of Technology, Toyohashi, Aichi 441-8580, Japan yoshinaga.tsukasa.es@osaka-u.ac.jp, zyzhang@ucla.edu, iida@me.tut.ac.jp

## Abstract

Previous experimental studies suggested that restraining the vocal fold vertical motion may reduce the coupling strength between the voice source and vocal tract. In this study, the effects of vocal fold vertical motion on source-filter interaction were systematically examined in a two-dimensional two-mass model coupled to a compressible flow simulation. The results showed that when allowed to move vertically, the vocal folds exhibited subharmonic vibration due to entrainment to the first vocal tract acoustic resonance. Restraining the vertical motion suppressed this entrainment. This indicates that the vertical mobility of the vocal folds may play a role in regulating source-filter interaction.

## 1. Introduction

In normal phonation, the vocal folds generally vibrate in an aerodynamically driven mode, in which vocal fold vibration is sustained by a near field interaction between the vocal folds and glottal airflow. However, vocal fold vibration may also be influenced by sub- and supra-glottal acoustic resonances (Ishizaka and Flanagan, 1972; Rothenberg, 1981; Fant, 1982; Zhang *et al.*, 2006a,b; Zañartu *et al.*, 2007; Titze, 2008; Lucero *et al.*, 2012; Kraxberger *et al.*, 2023; Zhang, 2023). This is particularly the case when the fundamental frequency *F*_0_ approaches the first resonance of either the subglottal or supraglottal tract. Under this condition, the vocal folds may vibrate in an acoustically driven mode, in which vocal folds are entrained to vibrate at a frequency close to the sub- or supra-glottal resonances (Zhang *et al.*, 2006a,b). More subtle effects of acoustic resonances are also observed when the fundamental frequency is not sufficiently close to an acoustic resonance [e.g., Titze and Story (1997) and Zhang (2023)].

Many factors can impact the degree of coupling between the vocal folds and the acoustic resonances of the sub- and supra-glottal tracts (the filter). One potential factor is the tendency of the vocal folds to vibrate in the vertical direction. In the experiments by Zhang *et al.* (2006a,b), their vocal fold model in an acoustically driven mode often exhibited a strong up-and-down, in phase motion. However, when the vertical motion was suppressed by applying a vertical restraint to the superior surface of the vocal folds, the vocal folds were induced into an aerodynamically driven mode, despite the tendency of the specific vocal fold model to vibrate in an acoustically driven mode. While the underlying mechanism is not clear, Zhang *et al.* (2006a) hypothesized that restraining the vertical motion of the vocal folds may reduce the coupling between the voice source and the sub- and supra-glottal acoustic resonances.

The goal of this study is to further explore this hypothesis and investigate the effects of restraining the vertical motion on source-filter interaction. While the vertical motion is inherently included in continuum models of vocal folds [e.g., Titze and Talkin (1979), Zheng *et al.* (2010), Yang *et al.* (2017), and Falk *et al.* (2021)], a modified two-mass model was used in this study, which allowed systematically suppressing the vertical motion of the vocal folds. There have been previous efforts in adding the vertical motion in lumped-mass models [e.g., Ishizaka and Flanagan (1977) and Adachi and Yu (2005)]. These studies showed that modeling the vertical motion is suitable for simulating soprano singing where the fundamental frequency is close to the first formant, at which large effects of vocal tract resonance are expected. However, the potential role of restraining the vertical motion in regulating source-filter interaction was not investigated in these previous studies.

In this study, a two-mass model that moves in both horizontal and vertical directions was coupled to a three-dimensional compressible flow. The use of the two-mass model enables us to gradually restrain the vertical motion while maintaining the same spring constants along the medial-lateral direction, thus isolating the potential effect of vertical motion on source-filter interaction.

## 2. Method

**Fig. 1.**
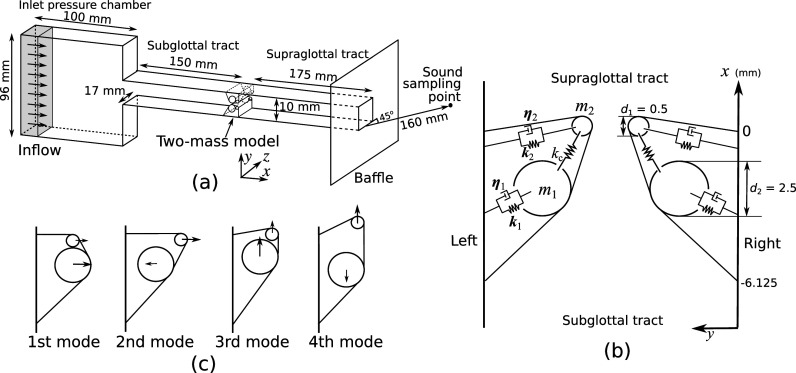
Overall flow channel and the two-mass model. The flow channel from the inlet pressure chamber to the vocal tract is shown in (a), and the two-mass model with horizontal and vertical motions is shown in (b). The four eigenmodes of the two-mass model for Q≥3 are depicted in (c). Note that the relative position of the masses in (b) was designed to illustrate the ability of the vocal folds to move vertically and does not represent the resting position used in this study.

The model geometry, including the vocal folds, subglottal, and supraglottal tract, is shown in Fig. 1(a). The sub- and supra-glottal tracts were simplified as rectangular channels of lengths 150 and 175 mm, respectively, based on general human male lengths. The subglottal tract was connected to an inlet pressure chamber with a volume of 100 × 96 × 17 mm^3^. The first three resonance frequencies of the subglottal tract were 500, 900, and 2525 Hz and 480, 1420, and 2380 Hz for the supraglottal tract. These frequencies were calculated by using the acoustics module of comsol multiphysics 6.1 (COMSOL, Inc., MA). Each vocal fold was modeled as two masses of cylinders. The vocal fold medial surface was formed by a smooth surface that is tangential to both cylinders, as in Pelorson *et al.* (1994). The masses were connected to the lateral wall by spring-dampers as shown in Fig. 1(b). Unlike the original two-mass model where the springs and dampers act in the medial-lateral direction only, in this study the springs and dampers had both a horizontal and vertical components, thus allowing the two masses to move in both directions. The two upper and lower masses were also connected with a coupling spring.

The equation of motions for two masses are

$$m_{1}\frac{d^{2}r_{1}}{dt^{2}}+\eta_{1}\circ \frac{dr_{1}}{dt}+k_{1}\circ r_{1}+k_{c}\left(r_{1}-r_{2}\right)=F_{1},$$ (1)

$$m_{2}\frac{d^{2}r_{2}}{dt^{2}}+\eta_{2}\circ \frac{dr_{2}}{dt}+k_{2}\circ r_{2}+k_{c}\left(r_{2}-r_{1}\right)=F_{2},$$ (2)

where $m_{i}$ is upper and lower masses (i = 1 and 2), $r_{i}=\left[r_{ix},\;r_{iy}\right]$ is the mass displacement vector, $k_{i}=\left[k_{ix},\;k_{iy}\right]$ is the spring stiffness vector, $F_{i}=[F_{ix},\;F_{iy}]$ is the external force vector on each mass, and $k_{c}$ is the coupling stiffness. The damping coefficients were calculated as $\eta_{1}=\left[0.1\times 2\sqrt{m_{1}k_{1x}},\;0.1\times 2\sqrt{m_{1}k_{1y}}\right]$ and $\eta_{2}=\left[0.6\times 2\sqrt{m_{2}k_{2x}},\;0.6\times 2\sqrt{m_{2}k_{2y}}\right]$. The symbol ∘ indicates the element-wise product. The coordinate *x* denotes the vertical axis (the superior-inferior directions), whereas *y* denotes the horizontal axis (the medial-lateral direction).

The masses were set as $m_{1}$ = 0.17 g and $m_{2}$ = 0.03 g based on Pelorson *et al.* (1994), and the spring constants were adjusted to $k_{1y}$ = 150 N/m, $k_{2y}$ = 40 N/m, $k_{c}$ = 20 N/m to obtain stable oscillations in this system. The vertical-to-horizontal stiffness ratio Q is defined so that $k_{ix}=Qk_{iy}$. In this study, the ratio Q was changed from 1.5 to 100 while the horizontal springs $k_{iy}$ were kept constant. In other words, the vertical spring constants were varied while other model parameters were kept constant, which allowed us to isolate effects of vocal fold vertical mobility. At rest, the glottis was closed at levels of both masses. Hence, the prephonatory glottal angle and minimal glottal gap were both 0. The effect of varying initial glottal opening was not considered in this study. During the oscillation, the inferior vocal fold surface angle and fold's vertical thickness varied with time, and the pressure forces were calculated over the surfaces at each time step.

The *in vacuo* eigenmodes of the two-mass model were calculated by solving the eigenvalue problem of Eqs. (1) and (2). The eigenfrequencies are listed in Table 1 and the eigenmodes for conditions with *Q* ≥ 3 are shown in Fig. 1(c). For *Q* ≥ 3, the first two modes exhibit motion in the medial-lateral direction only, whereas the last two modes exhibit motion in the vertical direction only. Reducing the *Q* value reduced the eigenfrequencies of the two modes with vertical motion. As a result, there is a crossover between the second and third eigenmodes as *Q* is increased from 2 to 3.

**Table 1.** Eigenmode frequencies in Hz of the two-mass model for different values of the vertical-to-horizontal stiffness ratio Q.

	Q							
	1.5	2	3	5	7	10	15	100
1st mode	153.2	153.2	153.2	153.2	153.2	153.2	153.2	153.2
2nd mode	187.0	215.3	229.2	229.2	229.2	229.2	229.2	229.2
3rd mode	229.2	229.2	262.7	337.7	398.6	475.5	581.3	1496.0
4th mode	262.9	292.8	345.3	431.8	503.8	595.8	723.7	1842.4

The airflow and sound around the oscillating vocal folds were calculated by solving the three-dimensional compressible Navier-Stokes equations with the finite difference method. By solving the equations with high-order-accuracy schemes (sixth-order-accuracy for space and third-order-accuracy for time), the flow and sound pressures were simultaneously solved for the turbulent flow around the vocal folds. This enables the full coupling of fluid-structure-acoustic interactions in the glottis. Flow turbulence was modeled with an implicit large eddy simulation with a tenth-order-accuracy spatial filter. To consider the moving wall boundaries of the vocal folds, we applied the volume penalization method as in the immersed boundary method (Yoshinaga *et al.*, 2022).

At the inlet of the pressure chamber, a constant pressure of 2000 Pa was imposed. At the outlet of the supra-glottal tract, a sound propagating space of 135 × 135 × 135 mm^3^ was added, and a non-reflecting boundary with a buffer region was applied as the outlet boundary conditions. The buffer region allows the propagating wave to attenuate before the wave reaches the outlet boundary (Freund, 1997). The minimum grid size near the vocal fold surface is 0.025 mm, and the total number of the numerical grids is approximately 131.4 × 10^6^. To compare the sound characteristics under the different Q values, we sampled the sound pressure at 160 mm from the mouth outlet [see Fig. 1(a)]. At the sampling point, we confirmed that the airflow velocity was small enough (<0.02 m/s) so that the pressure was dominated by acoustic pressure. The time step for time integration was set to 0.25 × 10^−7^ s with the Courant-Friedrichs-Lewy (CFL) number below 0.4 based on the speed of sound. The oscillation data were collected after obtaining stable oscillations, which often occurred after the first three initial oscillation cycles. The detailed methodology and verification for the computational accuracy were reported in Yoshinaga *et al.* (2022).

## 3. Results

**Fig. 2.**
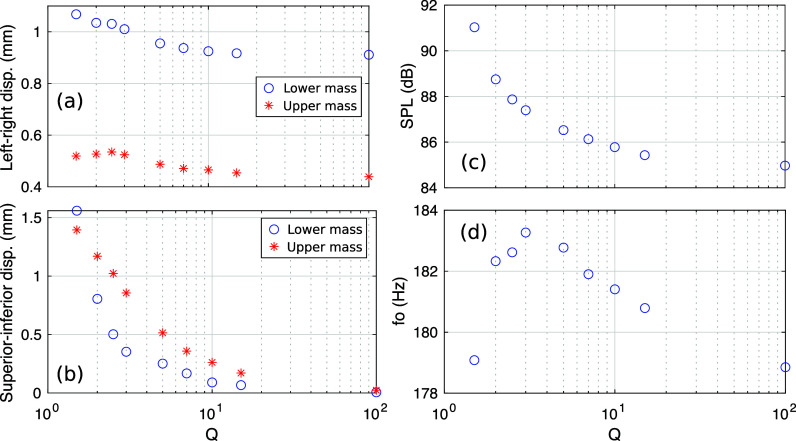
Output measures as a function of the vertical-to-horizontal stiffness ratio Q. Mean vocal fold displacements in the left-right (a) and superior-inferior (b) directions are plotted for each mass. The sound pressure level (c) was measured at 160 mm from the vocal tract outlet. The mean fundamental frequency (d) was extracted from the mass displacement.

The mean horizontal (medial-lateral) and vertical (inferior-superior) displacements, sound pressure level (SPL), and fundamental frequency *F*_0_ are plotted in Fig. 2 for different values of the vertical-to-horizontal stiffness ratio Q. With increasing Q (i.e., increasing vertical stiffness) while keeping the horizontal springs constant, the vertical displacements of both upper and lower masses decreased from around 1.5 mm at Q = 1.5 to almost 0 at Q = 100. At the same time, the horizontal (left-right) displacement only slightly decreased with increasing Q for both masses. The SPL at 160 mm from the mouth outlet also decreased with the increase in Q. The fundamental frequency varied slightly (less than 4 Hz), increasing first as Q was increased from 1.5 to 3, peaked at 183 Hz for Q = 3, then decreasing to 179 Hz with further increase in Q up to 100.

**Fig. 3.**
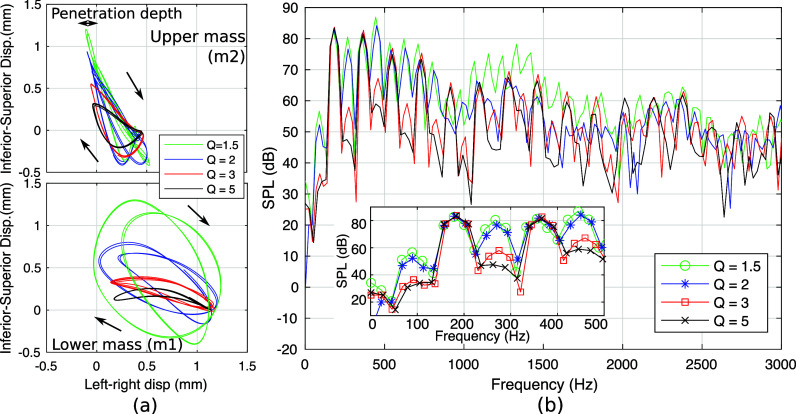
Motion trajectories of the two masses (a) and spectra of sound measured at 160 mm from the vocal tract outlet (b) for conditions with Q = 1.5 to 5. The inset shows the enlarged view of the spectra up to 500 Hz.

The motion trajectories of the upper and lower masses and corresponding far-field sound spectra for conditions Q = 1.5, 2, 3, and 5 are shown in Fig. 3. The displacement 0 indicates the initial position for each mass and left-right displacements below 0 indicates collision between left and right masses in Fig. 3(a). Two vibratory regimes were observed. For Q = 1.5 and 2, both masses vibrated with a torus trajectory. The sound spectra showed a peak at 450 Hz, approximately five times the subharmonic (5 × 90 = 450 Hz) in Fig. 3(b). This indicates a 2:5 entrainment frequency ratio between the fundamental frequency (180 Hz) and the first vocal tract resonance frequency (450 Hz), with the subharmonic frequency (90 Hz) being the difference of the two frequencies. These subharmonics suggest a likely rough voice quality (Berry *et al.*, 1996; Omori *et al.*, 1997). Formant peaks were also observed around 1340 and 2340 Hz, which were close to the estimated resonance frequencies of the rectangular supraglottal duct (480, 1420, 2380 Hz).

The subharmonics became much reduced for Q = 3 and disappeared when Q was further increased above 3. The two masses' trajectory changed to a limit cycle, and the first harmonic became the strongest. Increasing the Q also slightly reduced the penetration depths of the two masses (left-right displacements below 0; Fig. 3). These decreased depths probably resulted in the decrease in acoustic source magnitudes and far-field SPLs with increasing Q, as shown in Fig. 2(c).

**Fig. 4.**
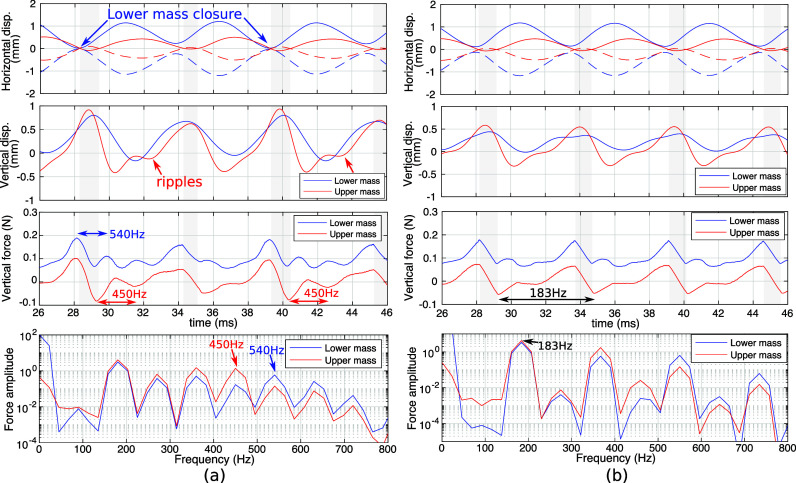
Horizontal and vertical mass displacements, vertical forces on the masses, and vertical force spectra for the case with Q = 2 (a) and Q = 3 (b). The solid and dotted lines in the horizontal displacements denote the left and right masses. The gray vertical bars indicate durations of complete glottal closure.

Figure 4 compares the horizontal and vertical mass displacements, vertical forces on the two masses, and vertical force spectra between the subharmonic oscillation at Q = 2 (left panel) and the regular oscillation at Q = 3 (right panel). The vertical forces were calculated by integrating the flow pressure over the corresponding vocal fold surface. The closed phases are indicated by gray vertical bars.

In the condition of subharmonic oscillation, the lower mass (*m*_1_) closed the glottis briefly once in every two cycles. Compared to the regular oscillation condition of Q = 3, the waveform of the upper mass vertical force in the subharmonic condition ( Q = 2) had strong ripples of about 450 Hz, which is close to the first resonance frequency of the supraglottal tract. This can be seen more clearly in the vertical force spectrum of the upper mass, which has local peak at 450 Hz. This in turn led to more noticeable ripples in the vertical displacement waveform of the upper mass (*m*_2_) compared to the waveform of the lower mass (*m*_1_). In contrast, the waveforms of the lower mass force had ripples at 540 Hz, which is close to the first subglottal resonance frequency (500 Hz). The vertical force spectrum of the lower mass also shows a peak at this frequency of 540 Hz. In other words, the subharmonic at 450 Hz appeared strongly only in the vertical force on the upper mass [indicated in Fig. 4(a) bottom], suggesting that the vocal fold vibration was entrained to the first vocal tract resonance instead of a subglottal resonance.

Kniesburges *et al.* (2016) reported subharmonic sound generation due to left-right glottal jet oscillations under certain supraglottal configurations. In our study, slight left-right glottal jet oscillation was also observed, particularly for conditions with subharmonic vocal fold vibrations. This can be observed in the supplemental multimedia movies, which show the flow field over two oscillation cycles for conditions Q = 2 (subharmonic oscillation) and Q = 5 (regular oscillation). It appears that the slight glottal jet oscillation was triggered by the subharmonic vocal fold vibration, rather than being of aerodynamic origin as in the study by Kniesburges *et al.* (2016).

## 4. Discussion

In this study, two vibratory regimes were observed as the vertical spring stiffness was varied while other model parameters were kept constant. For Q < 3, the vocal folds exhibited subharmonic vibration, due to a 2:5 entrainment between the fundamental frequency of vocal fold vibration (180 Hz) and the first vocal tract resonance frequency (450 Hz). The subharmonics gradually weakened with increasing Q. For Q ≥ 3, this entrainment to the vocal tract resonance was suppressed and the vocal fold vibration was changed to a regular vibration. Thus, increasing the vertical-to-horizontal stiffness ratio Q gradually suppressed the source-filter interaction and facilitated an aerodynamically driven mode of phonation.

Considering that the fundamental frequency varied within a small range (less than 4 Hz) as the Q was varied and both vibratory regimes covered the same range of *F*_0_, it is unlikely that the transition between the regimes was caused by a better frequency matching between the *F*_0_ and the first vocal tract resonance. In fact, increasing Q significantly increased the eigenfrequencies of the vertical-motion eigenmodes toward the first formant, which should have made it easier for the vocal folds to synchronize with the first vocal tract resonance at a 1:1 ratio at high Q values (e.g., Q = 5, 7, and 10). However, such 1:1 ratio entrainment with the acoustic resonance at the larger Q values was not observed in our study. In contrast, a 2:5 entrainment was observed only in conditions with much smaller Q values.

The observed changes in the degree of source-filter interaction were likely related to the ability of the vocal folds to move in the vertical direction, which may be more readily coupled to the acoustic back pressure from the vocal tract. In our study, this ability to vibrate in the vertical direction was controlled by the vertical-to-horizontal stiffness ratio Q. Increasing Q increased the vertical stiffness and suppressed the vertical motion of the vocal folds. This suppressed vertical motion effectively decoupled the vocal folds from the vocal tract, despite a potential *F*_0_-*F*_1_ matching at high Q values as mentioned above. On the other hand, the ability to move in the vertical dimension at small Q values allowed the vocal folds to synchronize with an acoustic resonance, even at a non-1:1 ratio, as observed in our study.

In this study, the subharmonics at small *Q* values occurred due to a 2:5 entrainment of vocal fold vibration to the vocal tract acoustics. It is reasonable to assume that for a different vocal tract geometry or fundamental frequency, the vocal folds may be entrained to the vocal tract in a different pattern, or not entrained at all. However, even in such conditions of weak source-filter interactions, we would expect that restraining the vocal fold vertical motion would still have a similar effect of weakening source-filter interaction. Future studies are required to clarify this issue.

This role of the vertical motion in regulating source-filter interaction is consistent with the result observed in our previous experimental studies (Zhang *et al.*, 2006a,b). These two studies showed that the vocal folds often exhibited larger vertical motion when entrained to acoustic resonances. Despite the tendency of their vocal fold model to vibrate in an acoustically driven mode of phonation, applying a vertical restraint, which suppressed the vertical motion of the vocal folds, allowed the vocal fold model to vibrate in an aerodynamically driven mode of phonation at a fundamental frequency independent of vocal tract resonances. The observation of the current study is also consistent with findings of a recent computational study (Zhang, 2023), which showed larger effects of source-filter interaction on the voice source in soft and long vocal folds. All else being equal, soft and long vocal folds have been shown to exhibit larger vertical motion than stiff and short vocal folds (Zhang, 2021), and thus presumably are more readily coupled to the acoustic resonance of the vocal tract.

As suggested in Zhang *et al.* (2006a), restraining the vertical motion of the vocal folds may be achieved by epilaryngeal manipulation through false vocal fold adduction, which may provide a natural vertical restraint on the true vocal folds. Activation of the thyroarytenoid and cricothyroid muscles may also restrain the vertical motion (Zhang, 2011). Further studies of such potential restraining mechanisms and their impact on source-filter interaction are needed, by utilizing finite element modeling and realistic laryngeal geometry [e.g., Wu and Zhang (2023)].

## 5. Conclusion

In this study, the effects of vocal fold vertical motion on source-filter interaction were investigated in a two-dimensional, two-mass model coupled to a compressible flow. The results showed that when vocal folds were allowed to move in the vertical direction, the vocal folds exhibited subharmonic vibration, with a 2:5 entrainment between the fundamental frequency and the first vocal tract resonance. This entrainment with the acoustic resonance was not observed when the vocal fold vertical motion was suppressed. These results indicate that restraining vocal fold vertical motion reduces the degree of source-filter coupling. Future studies are needed to further identify potential restraining mechanisms and their roles in regulating source-filter interaction.

## Supplementary material

See supplementary multimedia moviessupplementary multimedia movies for flow fields on the medial plane in the case with *Q* = 2 (SuppPub1.mp4) and *Q* = 5 (SuppPub2.mp4).

## Data Availability

The data that support the findings of this study are available from the corresponding author upon reasonable request.
